# The effects of biological sex on fatigue during and recovery from resistance exercise

**DOI:** 10.7717/peerj.20542

**Published:** 2026-01-08

**Authors:** Gregory Lee Nuckols, Chase Alexander Overpeck, Erik Daniel Hanson, Claudio Luiz Battaglini

**Affiliations:** Department of Exercise and Sports Science, University of North Carolina at Chapel Hill, Chapel Hill, NC, United States of America

**Keywords:** Strength training, Sex differences, Fatigability

## Abstract

**Background:**

Guidelines for resistance training prescription do not often consider sex as it relates to exercise prescription, despite its potential influence on responses to and adaptations following resistance training. If there are sex differences in the rate at which males and females fatigue during a resistance training session, or the rate at which they recover from resistance training between sessions, optimal resistance training volume may differ between the sexes. The purpose of this study was to investigate sex differences in fatigability and recovery from dynamic resistance exercise.

**Methods:**

Male and female subjects with at least one year of bench press experience (*N* = 21 males and 21 females) performed a fatigue protocol consisting of barbell bench press with 75% 1RM loads for sets of five repetitions, with 90 seconds between sets, until the point of concentric failure. Recovery was monitored for the subsequent 72 hours using subjective ratings of soreness for the pectoral muscles, triceps, and anterior deltoids, and estimated 1RM strength derived from load-velocity profiles.

**Results:**

The female subjects completed more reps during the fatigue protocol (females: 58.3 ± 27.3; males: 29.6 ± 10.6; *p* = 0.0001), but post-training soreness and recovery of estimated 1RM strength did not significantly differ between sexes (*p* > 0.05).

**Conclusion:**

Our results suggest that females fatigue slower than males during multiple sets of bench press yet appear to recover from training at the same rate in spite of completing a higher relative workload. Furthermore, the difference in performance during the fatigue protocol appears to be attributable to the female subjects recovering more quickly during the rest intervals, rather than fatiguing more slowly while performing each set.

## Introduction

Resistance training is commonly employed by strength and conditioning professionals to increase athletic performance, and by recreational athletes to improve strength and body composition. Meta-analyses have demonstrated that higher training volumes are associated with larger strength gains (Ralston et al., 2017) and greater muscular hypertrophy (Schoenfeld, Ogborn & Krieger, 2017). However, excess training volume can hinder strength gains (González-Badillo, Izquierdo & Gorostiaga, 2006) or muscular hypertrophy (Amirthalingam et al., 2017) if training volume at a given exercise intensity compromises positive adaptations by exceeding the body’s capacity to recover from exercise. Thus, resistance exercise prescription must balance the benefits of higher resistance exercise training volume and an individual’s capacity to recover from training to elicit the more positive adaptations. Many guidelines for resistance training prescription, including those of the National Strength and Conditioning Association (NSCA), do not consider sex as it relates to exercise prescription (Haff & Triplett, 2016), despite its potential influence on responses to and adaptations following resistance training. If there are sex differences in the rate at which males and females fatigue during a resistance training session, or the rate at which they recover from resistance training between sessions, optimal resistance training volume may differ between the sexes. Portions of this text were previously published as part of a preprint (Nuckols, 2019).

It is believed that anaerobic metabolism and the associated drop in muscle pH and accumulation of inorganic phosphate are important factors contributing to the development of performance fatigability (Enoka & Duchateau, 2016) during resistance exercise (Allen & Trajanovska, 2012; Metzger & Moss, 1990; Westerblad, Allen & Lännergren, 2002). The anaerobic fatigue induced by resistance training is influenced by muscle blood flow. Intense muscular contractions can partially occlude arteries (Hunter & Enoka, 2001), limiting blood flow and oxygen delivery. Consequently, anaerobic glycolysis must increase to match the energetic demands of exercise (Russ & Kent-Braun, 2003), leading to a drop in pH and accumulation of inorganic phosphate. These factors attenuate Ca^2+^ release from the sarcoplasmic reticulum, decrease the affinity of troponin C for Ca^2+^, and interfere with cross bridge cycling, thus decreasing muscle force (Allen & Trajanovska, 2012; Metzger & Moss, 1990; Westerblad, Allen & Lännergren, 2002; Wan et al., 2017). Furthermore, group III and IV muscle afferents, which can inhibit central motor drive, are also sensitive to ischemia, increased intramuscular pressure, and fatigue-associated metabolites (Hunter, 2009; Laurin et al., 2015). Since females have muscles with less cross-sectional area than males, on average (particularly in the upper body), they experience less arterial occlusion during forceful contractions, possibly decreasing the rate of fatigue development during resistance exercise (Hunter, 2014).

Neuromuscular recovery takes longer to accomplish in the presence of higher muscle damage (Byrne, Twist & Eston, 2004). This is illustrated by the fact that trained lifters recover their strength after training faster than untrained lifters, in part due to the collection of adaptations known as the repeated bout effect, which works to limit muscle damage (Hyldahl, Chen & Nosaka, 2017). It is commonly believed that females experience less muscle damage than males after resistance exercise, and females repair muscle damage faster than males. Higher estrogen levels have been shown to attenuate muscle damage and accelerate muscular regeneration (Enns & Tiidus, 2010); however, this is far from a unanimous finding in the literature. Much of the research demonstrating the protective effects of estrogen has come from animal models, but human studies do not consistently support this effect (Hubal & Clarkson, 2009; Macchi et al., 2024).

There is a considerable amount of research examining sex differences in acute fatigability during resistance exercise (Hunter, 2014; Hunter, 2016). In general, females appear to fatigue slower than males during resistance exercise. The primary mechanism is believed to involve greater preservation of muscle blood flow due to lower levels of muscle mass (Hunter & Enoka, 2001), but other potential reasons for lower fatigability in females include a greater relative muscle area occupied by type I muscle fibers (Hunter, 2014; Nuzzo, 2024), a lower reliance on anaerobic metabolism during submaximal exercise (Bunt, 1990; Hackney, 1999; Lundsgaard & Kiens, 2014; McCracken, Ainsworth & Hackney, 1994), and greater vasodilation and hyperemia during resistance exercise, in part due to an attenuated metaboreflex response (Parker et al., 2007; Ettinger et al., 1996). Most of the studies examining sex differences in fatigability during resistance exercise involve the use of submaximal isometric contractions, which is not reflective of typical practices in strength and conditioning. As such, less is known about sex differences in fatigability during dynamic, isotonic resistance exercise. Of the studies examining sex differences in fatigability during dynamic, isotonic resistance exercise, most confirm that females are less fatigable (Flanagan et al., 2014; Lanning et al., 2017; Maughan et al., 1986; Ribeiro et al., 2014; Senefeld et al., 2013; Yoon et al., 2015; Hulmi et al., 2025), while others find that males and females fatigue at similar rates (Clark et al., 2003; Mendonca et al., 2018; Pincivero, Coelho & Campy, 2004; Reynolds, Gordon & Robergs, 2006). Only one study employing a multi-set protocol has found that males are less fatigable than females (Monteiro et al., 2017).

There is less research examining sex differences in recovery from resistance exercise. Several studies have examined sex differences in post-exercise muscle soreness and sex differences in indirect markers of muscle damage, such as creatine kinase. Some find that males and females either experience similar soreness and elevations in markers of muscle damage (Benini et al., 2015; Flores et al., 2011), while others indicate that females experience less soreness and smaller elevations in markers of muscle damage (Amorim et al., 2014; Baggett, 2015; Wolf et al., 2012). Studies examining sex differences in recovery of neuromuscular performance remain scarce. One study found that females recovered 1RM strength sooner than males after a 5RM testing session (Judge & Burke, 2010), while another study found that training volume performed during a full-body workout was similarly suppressed 24-hours after resistance training in both males and females (Baggett, 2015). Finally, a more recent study found that isokinetic concentric knee extension torque and countermovement jump height were suppressed to a greater extent in females 24 hours after a squat training (Davies, Carson & Jakeman, 2018). A recent review noted that recovery following resistance training tended to be similar between sexes in most contexts, but noted that females have been found to display less fatigue and may recover more rapidly in certain contexts (Amdi et al., 2025). However, it also noted that there is a dearth of studies comparing males and females with comparable levels of relative strength and technical proficiency (Judge & Burke, 2010; Häkkinen, 1993). Thus, it remains unclear if males and females of similar training status recover from resistance training at different rates.

In strength and conditioning contexts, resistance exercise is generally performed with dynamic, isotonic exercises, and is typically not performed to failure on every set, as training to failure does not seem to enhance strength gains (Davies et al., 2016) and may increase injury risk (Nóbrega & Libardi, 2016). Most of the literature examining sex differences in fatigability and recovery, summarized by Hunter (2014), has either used isometric or isokinetic exercise, or has employed training protocols where all sets are performed to failure. This study seeks to determine if potential sex differences exist in fatigability during resistance training and recovery from resistance exercise using a protocol that is more reflective of current practices among strength and conditioning professionals (*e.g.*, multiple sets, with most sets not performed to the point of failure).

Therefore, the primary purpose of this study was to determine if males and females fatigue at different rates during a single fatiguing resistance exercise session, as assessed by total reps completed when performing submaximal sets of resistance exercise until the point of failure. The secondary purpose was to determine if males and females recover at different rates following a single training session, as assessed by the recovery of estimated relative 1RM strength (estimated 1RM as a percentage of their unfatigued estimated 1RM), and the attenuation of muscle soreness following the fatigue protocol. We hypothesized that females would complete a greater number of reps during the fatigue protocol. We also expected that females would recover faster than males when exposed to a given workload. Therefore, contingent upon our first hypothesis, we also hypothesized that (a) if our first hypothesis was incorrect (*i.e.,* if males and females completed a similar number of reps during the fatigue protocol), females would recover more quickly than males, but (b) if our first hypothesis was correct (*i.e.,* if females completed more reps than males during the fatigue protocol), there would be no significant sex differences in the rate of recovery from the fatigue protocol.

## Materials & Methods

This study utilized a quasi-experimental design with parallel groups. Both groups underwent the same intervention and testing. To be included in the study, the subjects must have had at least one year of consecutive bench press experience and be 18–35 years old. Subjects were free of any injury within the past six months that would have precluded them from safely participating or putting forth their best effort during maximal exercise. Furthermore, female subjects were pregnancy tested to verify that they were not pregnant. Due to the prevalence of hormonal contraceptive usage in the target population, hormonal contraceptive usage was not an exclusion criterion for female subjects. Of the 21 female subjects completing the study, 14 used hormonal contraceptives, and seven did not. Subjects were asked to refrain from any upper body resistance exercise beginning 48 h before their first visit to the lab, and continuing until the completion of all testing sessions.

### Ethical statement

All procedures were conducted in accordance with principles set out in the Declaration of Helsinki and approved by the University of North Carolina at Chapel Hill Institutional Review Board (IRB #18-1635).

### Procedures

The study included six visits to the lab, over a span of 8–11 days. The first two visits involved baseline assessments, the third visit was for a fatiguing resistance exercise protocol, and the final three visits were to monitor recovery following the resistance exercise protocol. A summary of the timeline of the study visits and associated events are presented in Table 1.

### Visits 1 and 2: baseline assessments

On a subject’s first visit, they were informed about the study protocol, screened for eligibility, and asked to provide written informed consent. All subjects provided urine samples for all visits to verify that they were adequately hydrated determined by urine specific gravity assessed *via* refractometer (TS Meter, American Optical Corp., Keene, NH, USA). Urine specific gravity was required to be between 1.00 and 1.02 for all testing visits. A urine pregnancy test was also administered to females during the first visit (due to the potential radiation from body composition testing) using strips that detect elevations of luteinizing hormone (Clinical Guard, Atlanta, CA, USA). A calibrated balance beam scale with height rod (Detecto, Webb City, MO, USA) was used to assess height and body mass. All subjects underwent a DEXA scan using a Discovery W Dual Energy X-ray Absorption (DEXA) scanner (Hologic, Inc., Bedford, MA, USA).

**Table 1:** Timeline of study events summary.

**VISIT 1**	**VISIT 2** **(48–72 hours** **after visit 1)**	**VISIT 3** **(48–72 hours** **after visit 2)**	**VISITS 4–6** **(24 hours** **after each visit 3–5)**
• Eligibility & Consent • Hydration & Pregnancy Test (if applicable) • Anthropometrics • DEXA Scan • Sleep, Stress, Training, and Dietary Questionnaires • 30–80%1RM Load-Velocity Assessment • 1RM Assessment	•Hydration Test • 30–80%1RM Load-Velocity Assessment • 1RM Assessment • 75%1RM Reps-to-Failure	• Hydration Test • Pre-exercise 30–80%1RM Load-Velocity Assessment • Pre-exercise lactate • Fatiguing Resistance Protocol • Post-exercise lactate • Post-exercise (acutely fatigued) 30–80%1RM Load-Velocity Assessment	• Hydration Test • 30–80%1RM Load-Velocity Assessment • Sleep & Soreness Questionnaires • Repeat Every 24 Hours for Visits 5&6

Lastly, subjects filled out questionnaires regarding their sleep, stress., training history, and dietary intake. Sleep duration and quality were estimated using the Pittsburgh Sleep Quality Assessment (PSQI; Chronbach’s *α* = 0.83; typical range for normal sleepers = 0–5) (Buysse et al., 1988). Stress levels were estimated using the Perceived Stress Questionnaire (PSQ; reliability: *r* = 0.84–0.86; low stress = 0–13; normal stress = 14–26; high stress = 27–40) (Cohen, Kamarck & Mermelstein, 1983). Aspects of the subjects’ training histories such as experience, typical bench press training volume and intensity, and goals were assessed *via* questionnaire. Dietary intake was assessed *via* 3-day food logs to be filled out over the course of the study. Subjects were instructed how to fill out the food logs, including portion sizes and brand names whenever possible, and asked to record two weekdays and one weekend day. This data was collected to help with the interpretation of the results if there were large differences in recovery rate to ensure those differences were not due to drastic sex differences in nutrient intake.

Following pre-testing assessments, each subject began a 5-minute self-selected warm-up of stretching and/or light calisthenics in preparation for the determination of their one-repetition maximum (1RM) bench press. After the warm-up, the subject was asked to estimate their 1RM bench press. 1RM bench press testing was performed on a standard bench press (Life Fitness, Rosemont, IL, USA) using a standard 20.4 kg Olympic barbell (Rogue Fitness, Columbus, Ohio, USA) and commercial-quality weight plates (Rogue Fitness, Columbus, Ohio, USA). The initial load used for testing was 30% of estimated 1RM if possible, or 15 kg if 30% of estimated 1RM was less than 15 kg. An additional 15 kg Olympic barbell (Eleiko, Halmstad, Sweden) was offered in this scenario.

Load was increased in increments of 10% of estimated 1RM until reaching approximately 80% of estimated 1RM. At this threshold, smaller load increases took place until a 1RM was attained in five or fewer attempts. Subjects rested for 2–3 min between each warm-up set and 2–5 min between each 1RM testing attempt until a 1RM was achieved. The shorter end of this interval (2–3 min) was only allowed between the first and second 1RM attempts during the first testing visit if it was obvious that the subject significantly underestimated their 1RM. Subjects performed three repetitions per set until reaching 80% of their estimated 1RM and performed single repetitions with all loads above 80% of estimated 1RM to minimize fatigue, which could hinder 1RM performance. For a repetition to be counted, the subject’s shoulders and gluteal region needed to maintain contact with the bench while their feet remained on the floor throughout the repetition. The bar started at full elbow extension, was lowered until it contacted the chest, paused briefly, and pressed until full elbow extension was attained. The subjects were instructed to control the eccentric portion of each repetition, and to perform the concentric portion of each repetition as fast as possible. Bench press load-velocity profiles were assessed using the GymAware Power Tool (Kinetic Performance Technology, Canberra, Australia); the repetition with the highest mean concentric velocity in each set was recorded. This device has been shown to have excellent validity and reliability with the loads used to establish load-velocity profiles in this study (relationship with gold-standard measure *r* > 0.9; CV < 10%) (Banyard et al., 2017; Banyard et al., 2018).

The second testing visit occurred 2–4 days later and followed the same 1RM assessment protocol. The minimum for the interval was determined by a previous study from Judge & Burke (2010) which showed bench press performance returned to baseline after 48hrs of rest in both males and females following maximal strength testing. Visit 2 began with another 1RM bench press assessment, using the same procedures described for Visit 1. After completing the 1RM assessment, the subject rested for five minutes before beginning a reps-to-failure test. The bar was loaded with 75% of the highest 1RM attained during the first two visits, and the subject performed as many repetitions as possible until concentric failure was achieved, or until the subject was unable to complete another repetition while meeting the technique guidelines discussed above (namely, briefly pausing the bar on their chest, and maintaining gluteal contact with the bench). The purpose of assessing strength endurance during a single set to failure was to ascertain whether any sex differences in performance during the fatigue protocol (during visit 3) were due to differences in single-set strength endurance, or due to differences in fatigue accumulation during the protocol independent of single-set strength endurance.

The subjects’ best bench press 1RM was scored using a formula developed by the International Powerlifting Federation for the purpose of comparing strength performances between lifters of different body weights and between the sexes (International Powerlifting Federation, 2019). The purpose of calculating this relative strength metric was to ascertain whether the male and female subjects in this study had comparable levels of strength and skill in the bench press. The aim was to recruit male and female lifters with similar degrees of skill and experience in the bench press, so that any potential differences in results could not be primarily attributable to differences in training status or bench press ability, rather than to differences between the sexes *per se*.

### Visit 3: fatiguing resistance exercise protocol

During the third visit, which occurred 2–4 days after visit 2, subjects completed a fatiguing exercise protocol. This visit began with a finger stick to assess pre-exercise lactate concentrations using a portable blood lactate analyzer (Lactate Plus, Sports Resource Group, Hawthorne, NY, USA). Following a 5-minute self-selected warm-up, the subjects completed six sets of bench press, with loads increasing from 30%1RM to 80%1RM in 10% increments, performing three repetitions at each load with 2–3 min between sets. This protocol was used as a load-velocity assessment and treated as the subject’s baseline performance. After this, the load was decreased to 75% 1RM and sets of five repetitions with 90 s of rest were performed until the point of concentric failure. This protocol is similar to the fatigue protocol employed in a recent study by Hulmi et al. (2025). Five minutes after the end of the last set, post-exercise blood lactate was assessed. Ten min after the end of the last set, the subjects repeated the load-velocity assessment. Subjects were again instructed to complete the concentric phase with maximal intended velocity for all load-velocity assessments.

**Figure 1:**
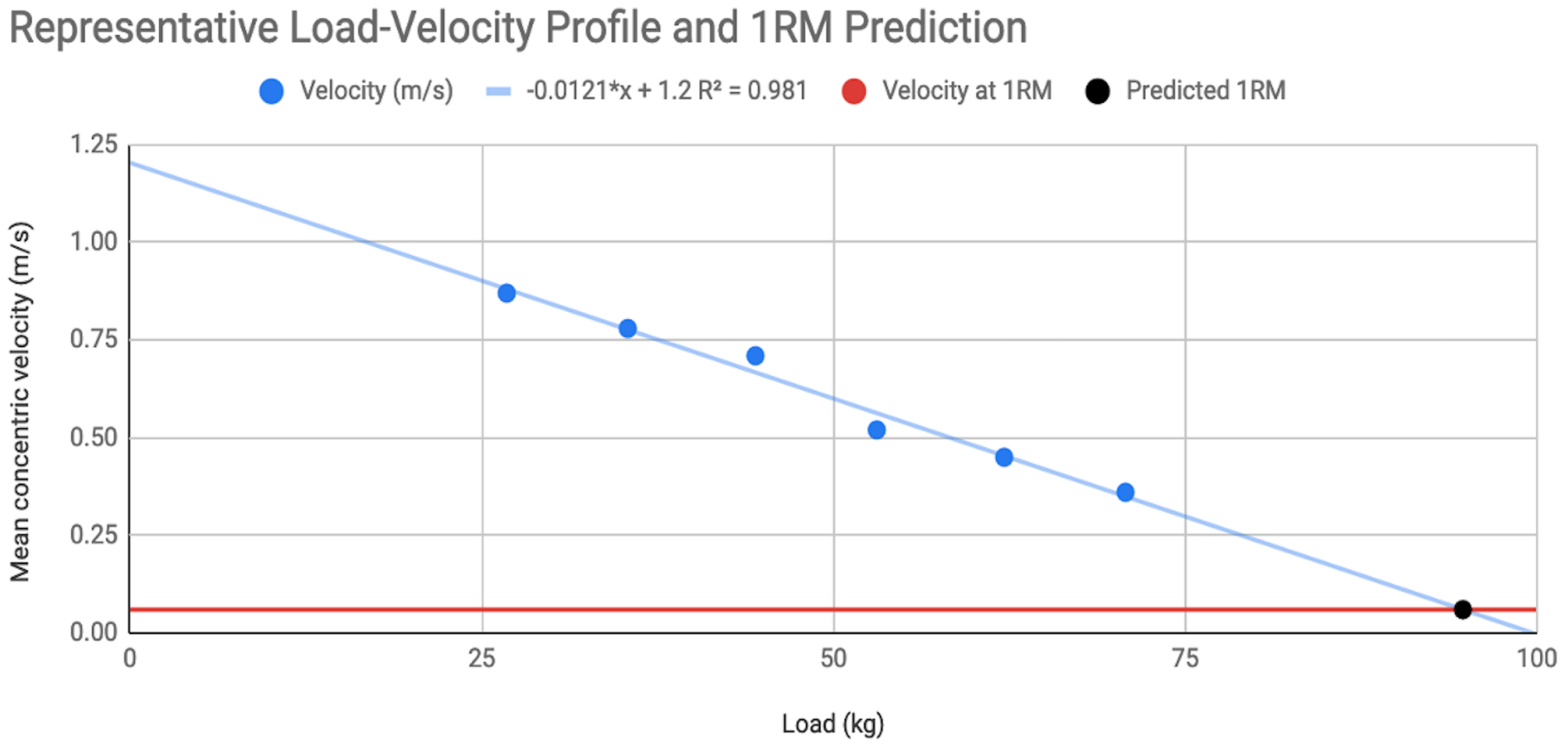
Representative load-velocity profile. In this representative load-velocity profile, mean concentric velocity was assessed at 30%, 40%, 50%, 60%, 70%, and 80% of 1RM (absolute loads: 26.8–70.8 kg). The best-fit linear trendline is shown. This subject had a known mean concentric velocity of 0.06 m/s when performing a 1RM. The trendline intersected a velocity of 0.06 m/s at a load of 94.7 kg, representing the subject’s predicted 1RM based on their load-velocity profile.

In visits 3–6, subjects’ load-velocity profiles were used to estimate their bench press 1RM, in order to assess acute decrements in strength performance, and recovery of strength over the next 72 h. To estimate 1RM strength, a linear trendline was fitted to each subject’s load-velocity profile, and 1RM was estimated by solving for load at the point where the load-velocity trendline intercepted the subject’s known velocity with 1RM loads (the mean concentric velocity recorded during the highest 1RM recorded during visits 1 and 2). Figure 1 shows a representative load-velocity profile, and the process used to estimate 1RM. Changes in predicted 1RMs assessed using this method are indicative of changes in maximal strength at a group level following a fatiguing resistance training session (Hughes et al., 2019). To assess recovery, subjects’ estimated 1RMs were expressed as a percentage of their unfatigued estimated 1RM (*i.e.,* the estimated 1RM obtained prior to the start of the fatigue protocol during visit 3).

### Visits 4–6: recovery assessments

For three consecutive days following the fatiguing exercise protocol, recovery from the fatiguing exercise protocol was assessed. The subjects began with a 5-minute self-selected warm-up. Daily load-velocity testing was conducted using the same protocol previously described. This protocol aimed to induce minimal additional fatigue and was used to gauge recovery of velocity output (with lower loads) and force output (with heavier loads). This was followed by questionnaires to assess sleep duration, sleep quality, and soreness of the pectoral muscles, triceps, and anterior deltoids. Sleep duration was self-reported in terms of hours the participant recalled sleeping the previous night. Subjective sleep quality and muscle soreness were self-reported on a 0–100 mm visual analog scale. For sleep quality, subjects were instructed that 0 and 100 represented the worst and best sleep imaginable, respectively. For muscle soreness, subjects were instructed to report how sore their pectoral muscles, triceps, and anterior delts felt while bench pressing, with 0 and 100 representing no soreness and the highest degree of soreness imaginable, respectively.

### Statistical analyses

All data were reduced and analyzed using JASP version 0.18.3 (JASP Team, 2024). Means and standard deviations or 95% confidence intervals are reported for all major variables. Total reps completed during the fatiguing exercise protocol was compared between sexes using Welch’s *t*-test.

Mean concentric velocity with low (50%1RM) and higher (80%1RM) load, and predicted relative 1RM from the subjects’ load/velocity profiles, were analyzed using 2 × 5 ANOVAs, with two levels of sex (male and female), and five time points (pre-exercise, post-exercise, 24-hours post, 48-hours post, and 72-hours post). Perceived muscle soreness was analyzed using a 2 × 3 ANOVA, with the same factors and the levels for sex, but only three time points (24-hours post, 48-hours post, and 72-hours post). A Greenhouse-Geisser correction was applied to all ANOVAs to limit the false discovery rate. If a significant group × time interaction was detected by any of the ANOVAs, a Bonferroni post hoc test was used to identify those interactions.

All other data collected (training histories, sleep, stress, *etc*.) were only used for exploratory analyses, descriptive purposes, or sensitivity analyses to ensure that any significant findings remain significant after covarying for other factors that could conceivably influence fatigability or post-exercise recovery. One exploratory analysis returned potentially interesting results, so it will be described in more detail here.

To explore whether observed sex differences in reps performed during the fatigue protocol were primarily attributable to different rates of fatigue accumulation during each set, or to different rates of recovery between sets, we calculated the mean velocity loss during each set (mean concentric velocity of the slowest rep in each set, minus mean concentric velocity of the fastest rep in the same set), and the mean velocity recovery between sets (mean concentric velocity of the fastest rep in one set, minus mean concentric velocity of the slowest rep in the previous set). Within-set velocity loss served as a proxy for fatigue accumulation, and velocity recovery between sets served as a proxy for the rate of recovery between sets. Within-set velocity loss and between-set velocity recovery were compared between sexes using independent samples t-tests.

To detect a group x time interaction in any of the recovery measures, a sample size of 40 subjects (20 per group) would be sufficient to detect a partial eta squared (*η*_p_^2^) effect size of 0.03, given a power of 0.8, an alpha of 0.05, and a correlation among within-subject measures of 0.5 (GPower ver. 3.1). For reps completed during the fatigue protocol, we anticipated that females would complete approximately 15 more reps than males, and we anticipated that the standard deviation within each sex would be approximately 10 reps based on our pilot testing. Given a sample size of 20 males and 20 females, we anticipated that the SE for both sexes would be approximately 2.2 reps, and the standard error for the difference between sexes would be approximately 3.2 reps.

Effect sizes were reported to contextualize results and group differences. For descriptive characteristics and the *t*-tests, effect sizes were reported as Cohen’s d (Trivial < 0.2, Small 0.2–0.5, Moderate 0.5–0.8, Large > 0.8). For correlations performed as exploratory analyses, effect sizes were reported as Pearson’s r. For ANOVAs, effect sizes were reported as partial eta squared, ${\eta }_{\mathrm{p}}^{2}$ (Small > 0.01, Moderate > 0.06, Large > 0.14).

## Results

### Participants

A total of 44 subjects (22 males and 22 females) were enrolled in the study. One male subject dropped out of the study after the second visit due to shoulder pain, and one female subject was found to not meet all inclusion criteria after the initial screening. Thus, the final sample consisted of 21 males and 21 females. Nine male subjects and five female subjects reported that their primary reason for bench pressing was to build muscle, two male and three female subjects reported that their primary reason for bench pressing was to improve strength endurance, and 10 male and 13 female subjects reported that their primary reason for bench pressing was to increase strength. Descriptive characteristics for the subjects can be seen in Table 2.

**Table 2:** Subjects characteristics and bench press training experience.

	Males (*N* = 21)		Females (*N* = 21)			
Variable	mean ± SD	Range	mean ± SD	Range	*p*-value	Effect size (d)
Age (years)	24.5 ± 4.1	(18.9–31.9)	24.0 ± 3.7	(19.7–34.2)	0.666	0.14
Weight (kg)^*^	83.4 ± 9.7	(66.9–108.4)	72.7 ± 10.3	(55.3–88.5)	0.001	1.07
Height (cm)^*^	174.5 ± 6.6	(162.5–187.6)	166.7 ± 8.5	(145.0–184.5)	0.002	1.03
BMI	27.4 ± 3.3	(22.7–35.3)	26.2 ± 3.8	(19.5–33.0)	0.278	0.34
DXA LBM (kg)^*^	65.7 ± 6.1	(55.5–78.4)	51.4 ± 6.6	(40.5–63.3)	<0.001	2.24
DXA body fat percentage^*^	19.9 ± 5.3%	(12.5%–31.4%)	28.1 ± 5.2%	(21.5%–38.5%)	<0.001	−1.56
DXA arm lean mass (kg)^*^	9.2 ± 1.2	(7.0–11.9)	5.8 ± 1.1	(3.7–7.8)	<0.001	3.01
PSQI seven component score	4 ± 2	(0–6)	3 ± 2	(0–10)	0.312	0.32
PSQ score	19 ± 6	(8–29)	20 ± 6	(6–28)	0.676	−0.13
Training years	7.2 ± 4.4	(1.5–17.0)	6.2 ± 5.3	(1.5–27.0)	0.542	0.19
Bench press years^*^	6.6 ± 4.2	(1.5–17.0)	4.1 ± 2.3	(1.0–10.0)	0.026	0.72
Bench press frequency per week	2.0 ± 0.9	(1.0–4.0)	1.6 ± 0.6	(1.0–3.0)	0.124	0.49
Bench press sets per week	9.8 ± 4.0	(4.0–20.0)	8.0 ± 4.3	(3.0–20.0)	0.165	0.44
Bench press sets of < 5 reps	23 ± 19%	(0%–90%)	35 ± 29%	(0%–100%)	0.108	−0.51
Bench press sets of 5–15 reps	74 ± 19%	(10%–100%)	62 ± 29%	(0%–100%)	0.129	0.48
Bench press sets of > 15 reps	3 ± 7%	(0%–25%)	3 ± 6%	(0%–25%)	0.728	0.11

**Notes.**

All data are mean ± SD (range). * Denotes a statistically significant difference between sexes (*p* < 0.05). A positive effect size (Cohen’s d) indicates a larger value for the male subjects. BMI, body mass index. DXA, Dual Energy X-ray Absorptiometry. LBM, Lean body mass. PSQI, Pittsburgh Sleep Quality Index. PSQ, Perceived Stress Questionnaire. The bench press sets < 5 reps, 5-15 reps, >15, and >5 reps are proportions of weekly sets within those respective rep ranges. Bench press frequency is sessions per week.

The males were taller, heavier, had more lean body mass, a lower body fat percentage, more arm lean mass, higher bench press 1RMs (*p* < 0.01 for all), and more years of bench press experience (*p* = 0.03). Age, sleep quality (PSQI scores), stress (PSQ scores), years of resistance training experience, all variables related to the subjects’ habitual bench press training (see Table 3), and scaled strength (IPF points) were not significantly different between sexes (*p* > 0.05). The males and females completed a similar number of reps during a single set to failure with 75% of 1RM in Visit 2 (females: 9.0 ± 2.0; males: 8.6 ± 1.7; t(39.035) = 0.743; *p* = 0.462; *d* = 0.23, 95% CI [−0.38–0.84]; Table 3).

**Table 3:** Results of strength and fatigability testing.

	Males (*N* = 21)		Females (*N* = 21)			
Variable	mean ± SD	Range	mean ± SD	Range	*p*-value	Effect size (d)
Bench press 1RM (kg)^*^	106.4 ± 27.8	(68.0–183.7)	57.0 ± 16.2	(30.4–87.1)	<0.001	2.17
Bench Press IPF points	432.3 ± 99.7	(256.4–628.7)	455.2 ± 99.0	(295.0–656.9)	0.459	−0.23
Reps completed with 75% to failure	8.6 ± 1.7	(6–11)	9.0 ± 2.0	(5–14)	0.462	−0.23
Reps completed during fatigue protocol^*^	29.6 ± 10.6	(13–54)	58.3 ± 27.3	(19–124)	<0.001	−1.39
Pre-training lactate (mmol/L)	1.4 ± 0.7	(0.5–2.9)	1.1 ± 0.4	(0.5–2.1)	0.081	0.56
Post-training lactate (mmol/L)^*^	7.2 ± 1.3	(4.9–10.1)	4.7 ± 1.2	(2.9–7.6)	<0.001	2.05
Change in lactate (mmol/L)^*^	5.8 ± 1.5	(3.6–9.1)	3.6 ± 1.3	(1.7–6.6)	<0.001	1.62

**Notes.**

All data are mean ± SD (range). * Denotes a statistically significant difference between sexes (*p* < 0.05). A positive effect size (Cohen’s d) indicates a larger value for the male subjects. IPF Points = bench press scoring system of the International Powerlifting Federation.

Dietary intake did not meaningfully differ between the male and female participants. For the male and female subjects respectively, reported energy intake was 28.0 *vs.* 29.1 kcal/kg, carbohydrate intake was 2.95 *vs.* 3.24 g/kg, fat intake was 1.13 *vs.* 1.13 g/kg, and protein intake was 1.70 *vs.* 1.59 g/kg.

Load/velocity profiles for the bench press were similar between sexes. Mean concentric velocity with loads from 30–80% of 1RM, velocity with 1RM loads (0.131 ± 0.044 m/s for males, *versus* 0.137 ± 0.070 m/s for females), and the average slope of the load/velocity curve (−0.123 ± 0.020 m/s per 10% of 1RM for males, *versus* −0.115 ± 0.020 m/s per 10% of 1RM for females) did not significantly differ between the male and female participants (*p* > 0.05 for all).

### Performance during the fatiguing resistance training session

**Figure 2:**
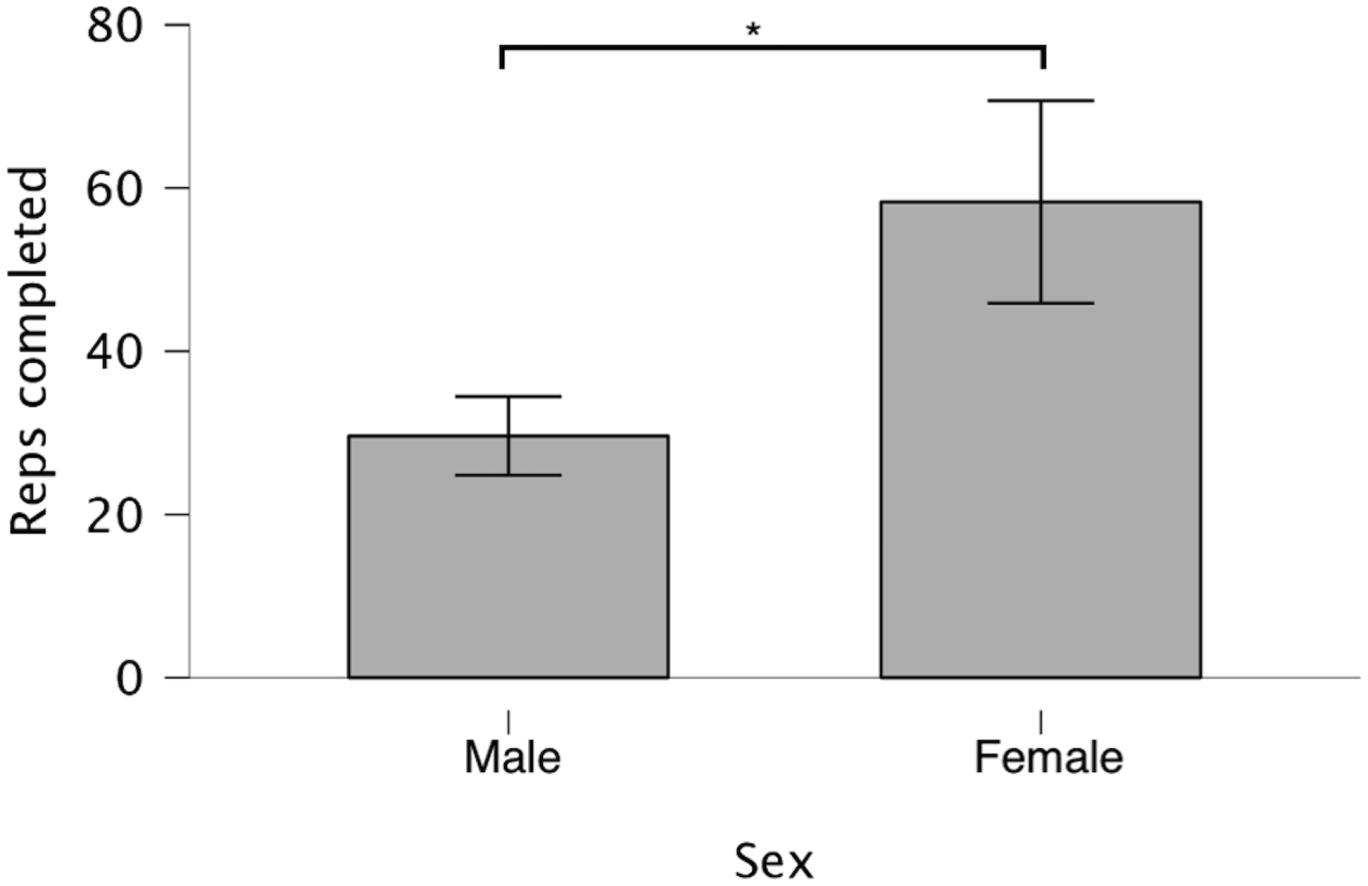
Total reps performed during fatigue protocol. Subjects performed consecutive sets of five bench press repetitions at 75%1RM with 90s rest intervals until concentric failure. Data are presented as mean and standard deviation for each sex with * denoting a significant (*p* < 0.05) difference between sexes.

To examine differences between the sexes in fatigability during a single resistance exercise session, we evaluated total reps when performing submaximal sets of resistance exercise until the point of failure. The females completed more total reps during the fatigue protocol in visit 3 (females: 58.3 ± 27.3; males: 29.6 ± 10.6; t(25.923) = 4.489; *p* = 0.0001; *d* = 1.39 95% CI [0.66–2.09]). The mean difference was 28.7 reps (95% CI [15.5–41.8]; Fig. 2). One outlier was detected in the female cohort (reps completed > Q3 + 1.5 × IQR). The difference was still significant with this data point removed (t(26.26) = 4.447; *p* = 0.0001; *d* = 1.40 (0.66–2.10)), though the mean difference decreased to 25.4 reps (95% CI [13.7–37.1]). The coefficient of variation for reps performed during the fatigue protocol was 47.8% for females and 35.8% for males, suggesting that in addition to large mean differences in fatigability between sexes, there is also considerable inter-individual variability within each sex.

In spite of completing more reps during visit 3, post-training lactate was lower in the females than the males, and the pre- to post-training increase in blood lactate was also smaller in females. Table 3 presents the performance and acute lactate data for the subjects. Post-exercise lactate levels, and pre- to post-training increases in blood lactate were not significantly associated with the number of reps completed during the fatigue protocol within each sex (*r*^2^ < 0.15; *p* > 0.1).

To assess if other variables contributed to reps completed during the fatigue protocol, all background training variables were individually tested as covariates in a multiple regression model also accounting for sex. Bench press sets per week was the only background training variable that was predictive of reps completed during the fatigue protocol (β = 1.705; *p* = 0.029) when also accounting for sex, such that subjects with higher habitual bench press training volume (sets per week) completed more total repetitions during the fatigue protocol.

### Recovery following a resistance training session

**Figure 3:**
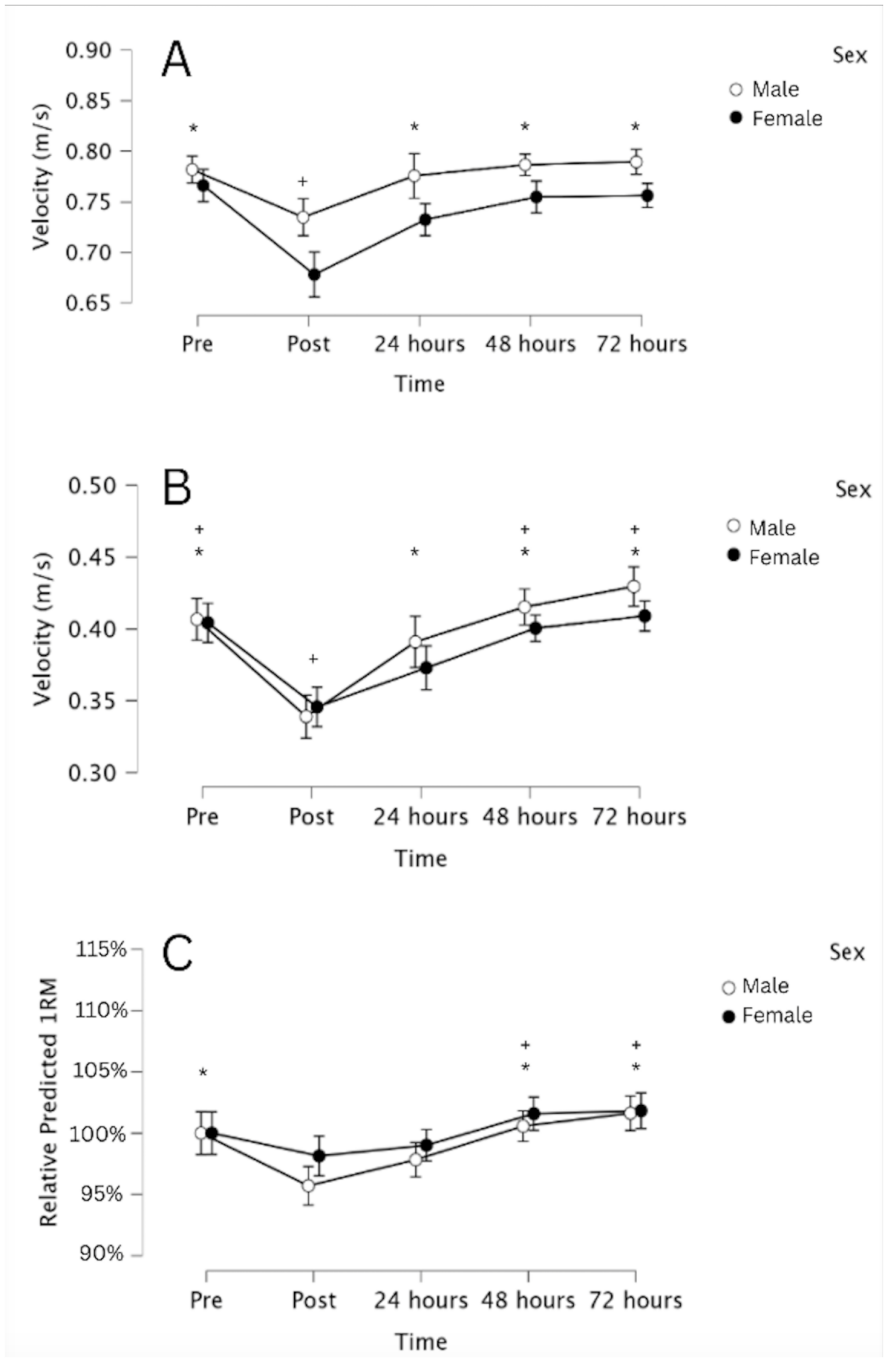
Decrements in neuromuscular performance and subsequent recovery of neuromuscular performance following a fatiguing bench press protocol. Changes in mean concentric velocity at 50% (A) and 80% of 1RM (B), and changes in relative predicted 1RM (C), calculated from individualized load-velocity profiles. Data are presented as mean and 95% CI for each of the five time points. * Denotes a significant (*p* < 0.05) difference from immediately post-exercise, and + denotes a significant (*p* < 0.05) difference from 24 h post-exercise.

Figure 3 depicts the recovery of velocity at 50% and 80% of 1RM, and recovery of normalized predicted 1RM strength in males and females. To examine differences between the sexes in neuromuscular recovery following a single resistance exercise session, we evaluated changes in mean concentric velocity with 50% and 80% of 1RM, and changes in estimated 1RM from load-velocity testing. A 2 × 5 (sex × time) ANOVA for predicted 1RM revealed significant time (F(2.772,110.880) = 11.842; *p* < 0.001; *η*_p_^2^ = 0.228) and sex (F(1,40) = 42.290; *p* < 0.001; *η*_p_^2^ = 0.514) effects, but no significant sex × time interaction (F(2.772,110.880) = 2.131; *p* = 0.105; *η*_p_^2^ = 0.051). When normalizing predicted 1RMs to the predicted 1RMs recorded prior to the fatigue protocol in visit 3, there was still a significant main effect for time (F(3.491,139.640) = 15.096; *p* < 0.001; *η*_p_^2^ = 0.274; Fig. 3), but no main effect for sex (F(1,40) = 0.837; *p* = 0.366; *η*_p_^2^ = 0.020) nor a sex × time interaction (F(3.491,139.640) = 0.907; *p* = 0.452; *η*_p_^2^ = 0.022) Normalized predicted 1RM was significantly lower 10 min post-exercise than pre-exercise (p_bonf_ = 0.01), and 48 h and 72 h post-exercise (p_bonf_ < 0.001). It was also significantly lower 24 h post-exercise than at 48 and 72 h post-exercise (p_bonf_ < 0.001). Changes in mean concentric velocity with 50% and 80% of 1RM followed similar trajectories. In both cases, there was a significant main effect for time (*p* < 0.001), but no significant effect for sex, and no significant sex × time interaction.

**Figure 4:**
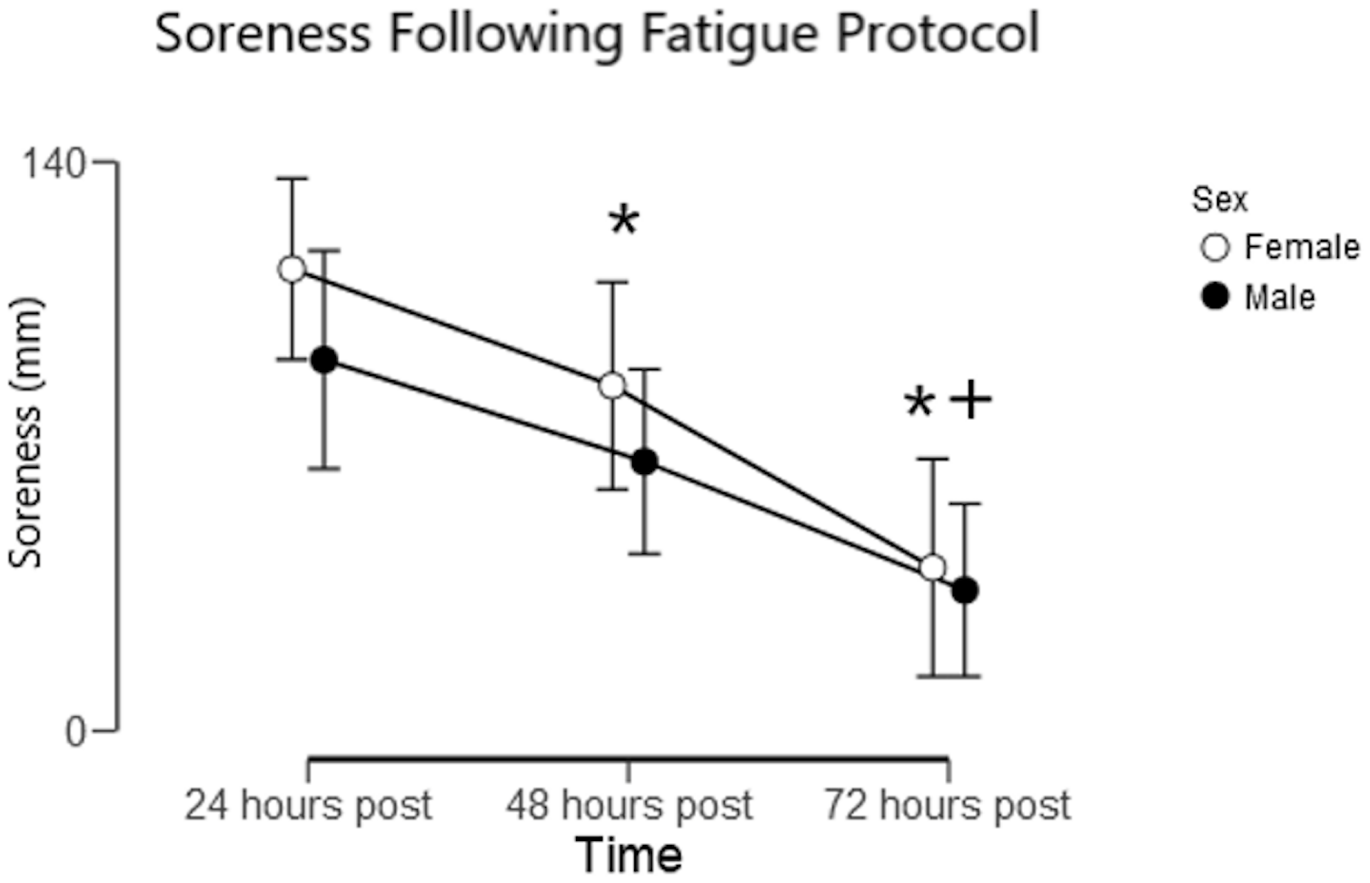
Muscle soreness following a fatiguing bench press protocol. Summed soreness of the pectorals, triceps, and anterior deltoids assessed using a 100 mm visual analog scale. Data are mean and 95% CI. * Denotes a significant (*p* < 0.05) difference from 24 h post-exercise, and + denotes a significant (*p* < 0.05) difference from 48 h post-exercise. There were no significant differences in perceived soreness between sexes at any point in time.

Multiple 2 × 3 (sex × time) ANOVAs for perceived muscle soreness found significant main effects for time for all three muscles examined (pectorals, triceps, and anterior deltoids) and for the summed soreness of all three muscles (*p* < 0.01 for all; *η*_p_^2^ = 0.524 for pectorals, 0.174 for triceps, 0.365 for anterior deltoids, and 0.531 for summed soreness). For the pectoral, anterior deltoids, and summed measures, soreness was significantly greater at 24 h post-exercise than at 48 and 72 h post-exercise, and significantly greater at 48 h post-exercise than at 72 h post-exercise (p_bonf_ < 0.05). For the triceps, there was no significant difference in soreness between 24 and 48 h post-exercise (p_bonf_ = 0.355), but soreness was significantly lower at 72 h post-exercise than at 24 and 48 h post-exercise (p_bonf_ < 0.05). There were no significant main effects for sex nor any significant sex × time interactions for the three muscles examined, nor for the summed soreness measures. Figure 4 provides a visual representation of the level of soreness following the fatigue protocol between the male and female subjects in this study.

For the male subjects, soreness 24 h post-training was moderately associated with reps completed during the fatigue protocol (*r* = 0.49; *p* = 0.025); however, there was not a significant association for the female subjects (*r* = 0.05; *p* = 0.81). The relative decrease in predicted 1RM 24 h post-training was not significantly associated with any baseline variables, dietary variables, or sleep the preceding night.

### Exploratory analysis

The sex difference in total reps performed in the current study was considerably larger than has been observed in prior research using protocols consisting of multiple sets to failure. Therefore, we performed an exploratory analysis on the load/velocity data gathered during the fatigue protocol to tentatively determine whether the difference in performance was primarily attributable to a slower rate of fatigue during exercise, or if it was primarily attributable to a faster rate of recovery during the inter-set rest intervals. Average velocity loss from the fastest rep to the slowest rep within each set of the fatigue protocol was similar between sexes (−0.152 ± 0.051 m/s for males and −0.157 ± 0.052 m/s for females; *p* = 0.40), but the recovery of velocity from the slowest rep of one set to the fastest rep of the next set was considerably greater in females (0.123 ± 0.045 m/s for males and 0.146 ± 0.053 m/s for females; *p* = 0.0001). As a result, velocity loss from the fastest rep of one set to the fastest rep of the subsequent set was considerably smaller for females (−0.029 ± 0.036 m/s for males and −0.012 ± .041 m/s for females; *p* = 0.0003). Thus, it appears that female lifters were primarily able to complete more reps during the fatigue protocol due to a faster rate of recovery between sets, rather than due to a slower rate of fatigue during each set. However, since this was an exploratory analysis that was not pre-specified prior to the start of data collection, it should be interpreted cautiously.

## Discussion

We hypothesized that the female lifters would be less fatiguable when performing multiple sets with limited rest intervals and, contingent upon our first hypothesis being correct, we hypothesized that the male and female lifters would recover from training at similar rates. The key findings of this study confirm both hypotheses: despite the male and female lifters completing a similar number of reps during a single set to failure with 75% of their 1RM, the females completed almost twice as many total reps during a fatigue protocol consisting of sets of five reps with 75% of 1RM and 90 s between sets, taken to the point of failure. Furthermore, despite females completing more reps during the fatigue protocol, recovery following the fatigue protocol was similar between the sexes, assessed *via* muscular soreness and predicted 1RMs from load-velocity profiles.

The finding that male and female lifters completed a similar number of reps to failure during a single set with 75% of their 1RM is consistent with previous literature (Flanagan et al., 2014; Maughan et al., 1986; Reynolds, Gordon & Robergs, 2006), and a recent meta-analysis (Nuzzo et al., 2024). Females may complete more reps during a single set to failure or have a longer time to task failure during isometric exercise when using low loads, but the difference in acute fatigability between the sexes is usually not significant with heavier loads (>70%1RM).

### Fatigability during a resistance training session

The fatigue protocol consisted of multiple sets of a fixed number of reps, stopping shy of failure on all sets except for the final set. Previous research has primarily used protocols consisting of a fixed number of sets with all sets taken to failure (Clark et al., 2003; Flanagan et al., 2014; Häkkinen, 1993; Häkkinen, 1994; Maughan et al., 1986; Monteiro et al., 2017; Miller et al., 1993; Pincivero, Coelho & Campy, 2004; Reynolds, Gordon & Robergs, 2006; Ribeiro et al., 2014; Salvador et al., 2005; Yoon et al., 2015), or it has prescribed a predetermined workload (Lanning et al., 2017; Mendonca et al., 2018; Senefeld et al., 2013) while assessing decrements in performance pre- to post-exercise. As discussed in the introduction, some of these studies suggest that female lifters are slightly less fatiguable than males, but most find no significant differences between the sexes. However, most individuals do not always train to failure, so it is also important to know whether males and females fatigue at different rates during submaximal training stopping shy of concentric failure. In contrast to prior studies that have exclusively involved training to failure, we observed very large differences in fatigability, with female lifters completing substantially more reps than the male lifters. However, only one prior study (Hulmi et al., 2025) has employed a fatigue protocol similar to the present study, and it also observed large sex differences in fatigability with considerable inter-individual variability within each sex. It is also interesting that these findings held true for different muscle regions as Hulmi et al. (2025) evaluated lower-body exercise and the present study evaluated upper-body exercise, suggesting that these sex differences may be generalizable rather than region-specific.

Our exploratory analysis suggests that the female participants were able to complete considerably more reps during the fatigue protocol primarily due to an enhanced rate of recovery between sets, compared to their male counterparts, rather than a diminished rate of fatigue during each set. If females recover faster between sets than males, the difference may be due to differences in metabolism and substrate utilization. Despite completing almost twice as many sets during the fatigue protocol, post-exercise lactate was substantially lower in the female subjects, suggesting less reliance on anaerobic glycolysis (lower lactate levels may also suggest less acidosis, which may contribute to the greater fatigue resistance observed in the female subjects). Other research has also found that female lifters experience smaller increases in blood lactate following resistance exercise (Szivak et al., 2013; Mochizuki et al., 2023). This may be related to differences in fiber type composition (Hunter, 2014) the effects of estrogen (Bunt, 1990; Hackney, 1999; Lundsgaard & Kiens, 2014; McCracken, Ainsworth & Hackney, 1994), or due to enhanced vasodilation and muscle perfusion (Parker et al., 2007). Additionally, the difference may be due to more efficient clearance of metabolic waste between sets in females. In a prior pair of studies using strength-matched males and females (Hunter et al., 2004a; Hunter et al., 2004b), time to task failure was the same between the sexes during continuous isometric contractions, but time to task failure was significantly greater for females during intermittent isometric contractions, even though the rest interval between contractions was only four seconds. If this was due to differences in fiber types or substrate utilization, one would expect a sex difference in time to task failure during continuous isometric contractions as well; the difference may instead be due to the females clearing the metabolic byproducts related to fatigue and force decrements—including hydrogen ions and free phosphate—more effectively than males, even during very limited rest intervals. More research is needed to understand the mechanisms underpinning the large difference in performance between the males and females in this study during the fatigue protocol. Monitoring RER to assess substrate utilization and using near infrared spectroscopy (NIRS) to examine local oxygen kinetics, especially during the recovery periods, would help with gaining insight into the physiology driving the differences. NIRS was used in Ansdell et al. (2019) and the researchers found that knee extensors of females preserve greater oxygen availability compared to males during intermittent, isometric exercise. There is still a lack of literature illustrating a similar trend during dynamic, isotonic exercise.

### Recovery following a resistance training session

Although females completed nearly twice as many reps as males during the fatigue protocol, recovery of estimated 1RM and post-training muscular soreness was similar between the sexes. This is a novel finding, as much of the prior research equated either the reps or sets performed by the subjects (Amorim et al., 2014; Baggett, 2015; Benini et al., 2015; Häkkinen, 1993; Häkkinen, 1994; Judge & Burke, 2010; Wolf et al., 2012). It is difficult to interpret this finding, since we are presently unable to test the counterfactual—would the females have recovered faster than the males if subjects of both sexes completed the same workload? The obvious assumption is that the females displayed greater recovery capacity than the males, since rates of recovery were similar despite the larger workload completed by the females. That would imply that the male lifters would have had a considerably longer time course for recovery if their workload was matched with the females in this study. However, multiple longitudinal studies have found that male lifters are capable of consistently employing and positively adapting to higher training volumes than those achieved by the female lifters in the study, suggesting that the male lifters in those studies did not exceed their capacity to recover (Radaelli et al., 2015; Enes, Souza & Souza-Junior, 2024; Brigatto et al., 2022; Schoenfeld et al., 2019). Furthermore, a recent systematic review reported that fatigue and recovery following resistance training are typically similar in males and females, even when workloads are matched (Amdi et al., 2025). Finally, within this study, total volume completed during the fatigue protocol was not associated with the time course of strength recovery; it was not associated with soreness in females and only moderately associated with soreness in males (*r* = 0.49). Furthermore, prior research suggests that training to failure (or closer to failure) has a large impact on the subsequent time course of recovery, but that squat and bench press performance may be entirely recovered 24 h after subjects performed 3–6 sets with multiple reps in reserve (Morán-Navarro et al., 2017; Pareja-Blanco et al., 2018). Thus, one possibility is that most of the recovery burden was primarily attributable to the final few sets of the fatigue protocol, in which the subjects were training very close to failure, rather than being due to the total volume accumulated (which would include many sets terminated far from failure for the subjects completing a large number of total set). If this is the case, the findings of this study would not suggest that female lifters have a greater recovery capacity than male lifters; rather, it would suggest that the fatigue protocol induced a similar amount of fatigue in both the male and female subjects due to a similar number of near-failure sets performed, despite the difference in total volume performed. Supporting this view, immediate decrements in performance from pre- to 10 min post-exercise were similar in the male and female subjects, suggesting that acute fatigue was similar between the sexes (*i.e.,* relative to the amount of measurable fatigue induced by the fatigue protocol, the male and female subjects recovered at similar rates).

Conversely, the more straightforward interpretation of the findings may be the correct interpretation: females recovered faster than males despite completing more total volume during the fatigue protocol, and thus, female lifters have a greater recovery capacity than males. If this interpretation is correct, the effect may be due, in part, to the effects of estrogens, which reduce muscle damage and aid in muscular recovery (Enns & Tiidus, 2010). However, as mentioned previously, these effects are most strongly established in animal models, and remain controversial in humans (Hubal & Clarkson, 2009). A second potential explanation relates to fiber type differences: females tend to have a greater relative type I fiber area than males (Nuzzo, 2024). Type I fibers are less susceptible to muscle damage (Stožer, Vodopivc & Križančić Bombek, 2020), and athletes with a greater estimated proportion of type I fibers may recover more quickly following fatiguing exercise (Lievens et al., 2020). However, this interpretation contrasts with the results of a study by Davies, Carson & Jakeman (2018). They found that males recovered faster after five sets of five squats with 80% of 1RM, followed by one set to failure, as assessed by concentric knee extension torque and countermovement jump height. The difference may be due to the choice of lower-body *versus* upper-body exercise. Ultimately, it remains unclear whether there are generalizable sex differences in recovery capacity following resistance training, and more research is needed on the topic. To improve upon the methods employed in the present study, future research could measure pressure-pain threshold to assess soreness (instead of relying on purely subjective rating), assess isotonic, concentric, eccentric, and isometric strength independently, and measure biomarkers related to stress, inflammation, and muscle damage (for example, cortisol, pro-inflammatory cytokines, C-reactive protein, creatine kinase, myoglobin, and cell-free DNA) to provide a more holistic view of the recovery process.

### Limitations

This study has numerous limitations. The only exercise assessed was the bench press, so the results of this study may not generalize to other exercises and muscle groups. The only biomarker data collected was blood lactate levels before and after the fatigue protocol—biochemical markers of stress, inflammation, and muscle damage would provide a more holistic understanding of the recovery process following exposure to a fatigue protocol similar to the one employed in this study. All subjects were in their 20s or early 30s, so it remains unclear if these results would generalize to teenage, middle-aged, or older lifters. Hormonal contraceptive status and menstrual cycle phase may have small effects on performance and recovery in female athletes, but they were not controlled in this study. Scheduling conflicts prevented some subjects from being tested at the same time of day in all sessions which could influence the results due to diurnal strength fluctuations. Subjects reported subjective feelings of soreness, but algometry would be capable of providing a more objective assessment of muscle discomfort. We were unable to assess muscle perfusion and oxygenation during exercise, so mechanistic explanations for our findings remain tentative and speculative. Finally, the use of dynamometry, EMG, and TMS would allow for more holistic assessments of neuromuscular recovery following a similar fatigue protocol.

## Conclusions

Despite completing a similar number of reps during a single set to failure at 75% of 1RM, females were able to complete approximately twice as many total reps during a fatigue protocol consisting of multiple sets terminated before failure. Recovery of muscle soreness and estimated 1RM following training was similar between sexes. In aggregate, these findings suggest that females engaged in resistance training can likely train the bench press with higher per-session relative training volumes than males while still recovering at approximately the same rate. Since this study used a fatigue protocol that differed considerably from prior research, further studies are warranted to confirm or refute these findings using similar protocols to optimize sex-based training practices.

Longitudinal studies are needed to determine the degree to which these findings can be used to inform resistance exercise program design in order to improve neuromuscular adaptations. However, if we assume that higher training volumes promote greater hypertrophy and larger increases in muscle strength when the lifter is capable of recovering higher volumes, and if we additionally assume that the individualized amount of training volume an individual lifter can adapt to is related to the workloads a lifter can handle in each workout, combined with their ability to recover between training sessions, we can tentatively conclude that female lifters may be able to benefit from higher bench press volumes than male lifters, on average. More concretely, we find that female lifters are capable of recovering faster than male lifters between sets, which suggests that coaches may be able to employ shorter rest intervals with their female athletes than their male athletes without compromising their female athletes’ ability to continue performing well within a workout. Finally, we found that single-session fatigability varies considerably both between sexes, and also within each sex. Coaches should therefore personalize rest times and workloads to match the capacities of their athletes.

## Supplemental Information

10.7717/peerj.20542/supp-1 Supplemental Information 1 Raw Data

## Additional Information and Declarations

### Data Availability

The following information was supplied regarding data availability: The raw data is available in the Supplementary File.
